# Unaltered Glutamate Transporter-1 Protein Levels in Aquaporin-4 Knockout Mice

**DOI:** 10.1177/1759091416687846

**Published:** 2017-01-01

**Authors:** Jacqueline A. Hubbard, Devin K. Binder

**Affiliations:** 1 Center for Glial-Neuronal Interactions, Division of Biomedical Sciences, University of California, Riverside, CA, USA

**Keywords:** coimmunoprecipitation, colocalize, EAAT2, physical interaction

## Abstract

Maintenance of glutamate and water homeostasis in the brain is crucial to healthy brain activity. Astrocytic glutamate transporter-1 (GLT1) and aquaporin-4 (AQP4) are the main regulators of extracellular glutamate and osmolarity, respectively. Several studies have reported colocalization of GLT1 and AQP4, but the existence of a physical interaction between the two has not been well studied. Therefore, we used coimmunoprecipitation to determine whether a strong interaction exists between these two important molecules in mice on both a CD1 and C57BL/6 background. Furthermore, we used Western blot and immunohistochemistry to examine GLT1 levels in AQP4 knockout (AQP4^−/−^) mice. An AQP4-GLT1 precipitate was not detected, suggesting the lack of a strong physical interaction between AQP4 and GLT1. In addition, GLT1 protein levels remained unaltered in tissue from CD1 and C57BL/6 AQP4^−/−^ mice. Finally, immunohistochemical analysis revealed that AQP4 and GLT1 do colocalize, but only in a region-specific manner. Taken together, these findings suggest that AQP4 and GLT1 do not have a strong physical interaction between them and are, instead, differentially regulated.

## Introduction

The excitatory amino acid transporters (EAATs) are a family of five membrane proteins that are responsible for clearing glutamate from the extracellular space. EAAT1 and EAAT2, also called glutamate aspartate transporter (GLAST) and glutamate transporter-1 (GLT1) respectively in rodents, are largely expressed by astrocytes. EAAT3 (termed excitatory amino acid carrier-1 in rodents) and EAAT4 are predominately found in neurons. Finally, EAAT5 is exclusively found in rod photoreceptor and bipolar cells in the retina (Kim et al., 2011). GLT1 is responsible for the majority of glutamate clearance in the forebrain (Danbolt, 2001). More recently, it was discovered that GLT1, although anchored to the membrane at synapses, can become untethered to perform rapid-activity-regulated surface diffusion to efficiently clear glutamate after synaptic activity (Murphy-Royal et al., 2015; Al Awabdh et al., 2016). In this manner, GLT1 can shape excitatory postsynaptic currents.

A few studies have found that GLT1 colocalizes with another important brain protein, aquaporin-4 (AQP4; Hinson et al., 2008; Lee et al., 2013; Yang et al., 2013; Mogoanta et al., 2014). A member of the aquaporin family of water channels, AQP4 and is responsible for bidirectional fluid exchange in response to changes in the osmotic gradient. AQP4 is the main water channel in the central nervous system (CNS) and, like GLT1, is found almost exclusively on astrocytes (Nielsen et al., 1997; Nagelhus et al., 2004; Hsu et al., 2011). AQP4 exhibits highly polarized expression with abundant levels found at sites of fluid transport, such as ventricular membranes, glia limitans, as well as pial and ependymal surfaces in contact with cerebrospinal fluid (Nielsen et al., 1997; Rash et al., 1998; Nagelhus et al., 2004; Oshio et al., 2004; Costa et al., 2007; Hubbard et al., 2015).

Since the generation of AQP4 knockout (AQP4^−/−^) mice (Ma et al., 1997), several different functions of AQP4 have been elucidated. It is now known that AQP4 plays a variety of roles in the brain, including (but not limited to) synaptic plasticity (Skucas et al., 2011; Scharfman and Binder, 2013), K^+^ buffering (Binder et al., 2006), and macromolecular diffusion in the brain extracellular space (Binder et al., 2004). Therefore, AQP4^−/−^ mice exhibit several deficits such as cognitive impairment (Skucas et al., 2011; Fan et al., 2013; Scharfman and Binder, 2013; Szu and Binder, 2016), perturbed K^+^ homeostasis (Strohschein et al., 2011; Haj-Yasein et al., 2015), and an enlarged extracellular space (Yao et al., 2008; Haj-Yasein et al., 2012).

Reduced levels of GLT1 protein have been reported in AQP4^−/−^ mice (Zeng et al., 2007; Wu et al., 2008; Chen et al., 2010; Li et al., 2012; Yan et al., 2013; Yang et al., 2013; Kong et al., 2014). Therefore, this expression and functional downregulation has been used to explain some of the impairments seen in AQP4^−/−^ mice. For example, downregulation of GLT1 has been hypothesized to play a role in synaptic plasticity and memory formation deficits in the AQP4^−/−^ mice (Li et al., 2012; Szu and Binder 2016). One study found an interaction between AQP4 and GLT1 in transfected HEK293 cells (Hinson et al., 2008). A physical interaction between AQP4 and GLT1, however, has never been confirmed in tissue from healthy, wild-type mice. Therefore, we performed coimmunopreciptation on both C57BL/6 and CD1 mice but found no AQP4-GLT1 precipitate. Furthermore, Western blotting and immunohistochemistry revealed no altered GLT1 protein expression or localization in AQP4^−/−^ mice compared with wild-type mice. Therefore, a physical interaction between AQP4 and GLT1 is unlikely, although a weak interaction between the two proteins cannot be ruled out.

## Methods

### Animals

All experiments were conducted in accordance with National Institutes of Health guidelines and were approved by the University of California, Riverside Institutional Animal Care and Use Committee (IACUC). Animals were housed under a 12-hr light/dark cycle with food and water provided *ad libitum*. CD1 and C57BL/6 wild-type and AQP4 knockout mice were used. All animals were 8 to 10 weeks old.

### Tissue Collection

Mice were euthanized with fatal plus (Western Medical Supply) and perfused with phosphate buffered saline (PBS), pH 7.4, with protease inhibitors (Roche). Whole brains were quickly removed from C57BL/6 wild-type (*n* = 6), C57BL/6 AQP4^−/−^ (*n* = 6), CD1 wild-type (*n* = 6), and CD1 AQP4^−/−^ (*n* = 5) mice. A separate subset of mice were euthanized, perfused, and hippocampal and cortex tissue was microdissected out from C57BL/6 wild-type (*n* = 7), C57BL/6 AQP4^−/−^ (*n* = 7), CD1 wild-type (*n* = 5), and CD1 AQP4^−/−^ (*n* = 5) mice. Harvested tissue was homogenized using the Bullet Blender (Next Advance) and protein concentration was quantified via Bradford assay. Protein was frozen at −80℃ until further processing.

For immunohistochemistry, C57BL/6 wild-type (*n* = 3), C57BL/6 AQP4 knockout (*n* = 3), CD1 wild-type (*n* = 3), and CD1 AQP4 knockout (*n* = 3) mice were transcardially perfused with ice-cold phosphate buffered saline (PBS), pH 7.4, followed by 4% paraformaldehyde, pH 7.4. Brains were quickly removed and postfixed in 4% paraformaldehyde overnight at 4℃ followed by 2 days of cryoprotection in 30% sucrose in PBS at 4℃. Brains were then frozen and stored at −80℃ until further use.

### Coimmunoprecipitation

Protein A agarose bead slurry (Roche) was equilibrated with 1% NP-40 substitute lysis buffer (50 mM Tris-HCl, pH 8; 150 mM NaCl; 1% NP-40 substitute). Protein samples (50 µg) were precleared by incubation with the equilibrated beads and then added to a new tube. Rabbit anti-AQP4 (Millipore ABN411), mouse anti-GLT1 (C-terminal polyclonal antibody (Rothstein et al., 1994)), normal rabbit IgG (Invitrogen 10500C), or normal mouse IgG (Invitrogen 10400C) was added to the samples, which were then incubated overnight under rotation at 4℃. The next day, protein A agarose beads were added to each sample and incubated for 3 hr under rotation at 4℃. Samples were centrifuged and supernatant removed (but saved). Beads with bound sample were then washed with 1% NP-40 lysis buffer. Finally, an SDS loading buffer was added to each sample, and samples were then boiled at 120℃ for 10 min to both denature the protein and separate it from the beads. All tubes were centrifuged and the supernatant was saved for Western blot.

### Western Blot

Protein was resolved by SDS-PAGE using 10% polyacrylamide gels and then transferred to a nitrocellulose membrane. Membranes containing coimmunoprecipitated (co-Ip) protein samples were probed with rabbit anti-AQP4 (1:1,000, Millipore ABN411) or rabbit anti GLT1 (1:2,500 of a C-terminal polyclonal antibody Rothstein et al., 1994). Membranes containing whole brain, hippocampal, or cortical protein for quantification were probed with rabbit anti-GLT1 (1:5,000 of a C-terminal polyclonal antibody, Rothstein et al., 1994) and mouse anti-β-actin (1:5,000, Sigma A1978) as an internal control. Bands were visualized using the Li-COR Odyssey Fc Western Imaging System. GLT1 lower bands were normalized to internal β-actin bands for quantification.

### Immunohistochemistry

Brains were thawed and cut into 50 µm sagittal sections using a cryostat (Leica CM 1950, Leica Microsytems, Bannockburn, IL) and slices were stored in PBS at 4℃. Two slices from each animal (*n* = 3 for each group) were used. Endogenous peroxidase activity was quenched by incubating slices in 3% H_2_O_2_ for 1 hr at room temperature. This was followed by a 1-hr blocking step with 5% normal goat serum in 0.1 M PBS. Slices were then incubated with primary antibody to AQP4 (1:200, Millipore ABN411) and/or GLT1 (1:200, C-terminal polyclonal antibody, Rothstein et al., 1994) in 0.3% Triton X-100 overnight at 4℃. After washing slices with PBS, sections were incubated with species-specific secondary antibody conjugated with Alexa 488 or 594 and a tyramide signaling amplification (TSA) kit (Molecular Probes/Invitrogen) for visualization. Slices were mounted in Vectorshield with 4′,6-diamidino-2-phenylindole (Vector Laboratories) and confocal images were taken using the Leica SP5 inverted microscope.

### Statistical Analysis

Statistical analysis was performed in GraphPad Prism using an unpaired *t* test. All error bars are presented as the mean ± *SEM*.

## Results

### AQP4 and GLT1 Do Not Co-Ip

**Figure 1.**
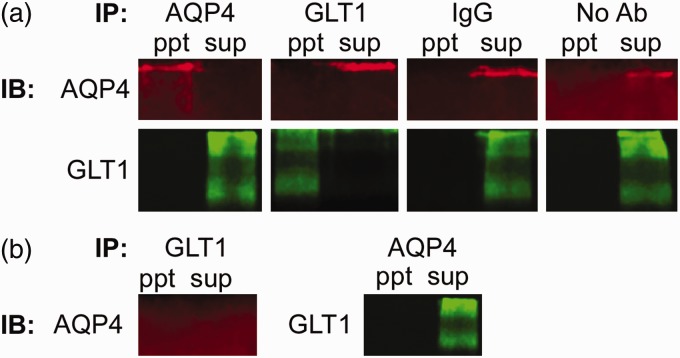
Lack of coimmunoprecipitation between glutamate transporter-1 (GLT1) and aquaporin-4 (AQP4) in C57BL/6 mice. (a) Coimmunoprecipitation of AQP4 and GLT1 in C57BL/6 wild-type mice. Representative images of the precipitants (ppt) AQP4, GLT1, normal IgG (mouse or rabbit), and no antibodies immunoprecipitated (IP) and either AQP4 (red; ∼150 KDa band) or GLT1 (green; ∼120–160 KDa bands) immunoblotted (IB). In addition, the supernatant (sup) from each sample was also run to represent the unbound sample. (b) Co-immunoprecipitation of GLT1 and AQP4 in C57BL/6 AQP4^−/−^ tissue. After GLT1 was IP, AQP4 was not detected in either the IBpptor sup. GLT1, however, was detected in the sup, but not theppt, after AQP4 was immunoprecipitated.

**Figure 2.**
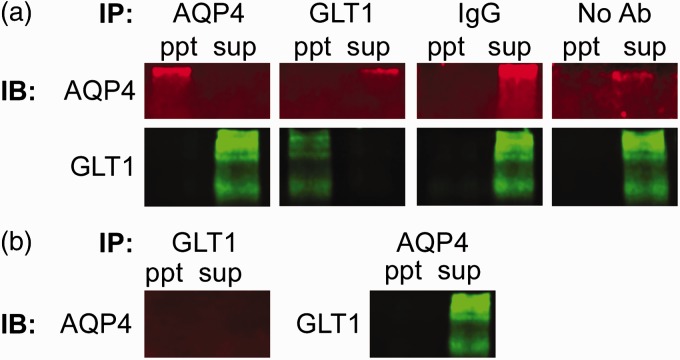
Lack of coimmunoprecipitation between glutamate transporter-1 (GLT1) and aquaporin-4 (AQP4) in CD1 mice. (a) Coimmunoprecipitation of AQP4 and GLT1 in CD1 wild-type mice. Representative images of the precipitants (ppt)AQP4, GLT1, normal IgG (mouse or rabbit), and no antibodies immunoprecipitated (IP) and either AQP4 (red; ∼150 KDa band) or GLT1 (green; ∼120–160 KDa bands) immunoblotted (IB). In addition, the supernatant (sup) from each sample was also run to represent the unbound sample. (b) Coimmunoprecipitation of GLT1 and AQP4 in CD1 AQP4^−/−^ tissue. After GLT1 was IP, AQP4 was not detected in either the IBpptor sup. GLT1, however, was detected in the sup, but not theppt, after AQP4 was immunoprecipitated.

Co-immunoprecipitation was used to determine whether a physical interaction exists between AQP4 and GLT1. We found, however, no resulting precipitate in whole brain tissue from C57BL/6 (Figure 1) or CD1 (Figure 2) mice under the conditions of 1% NP-40 lysis buffer. Both proteins were only detectable in the supernatant, representing the unbound population of protein. As positive controls, we determined that both AQP4 and GLT1 can be successfully immunoblotted after immunoprecipitation. When no antibodies or only mouse or rabbit IgG were immunoprecipitated, neither AQP4 nor GLT1 were detected with immunoblotting. In addition, no detectable AQP4 band in either the immunoprecipitated sample or the supernatant was detected after immunoprecipitation of GLT1 in AQP4^−/−^ tissue. When AQP4 was immunoprecipitated out from AQP4^−/−^ tissue, GLT1 was only detectable in the supernatant (unbound) sample, as expected. Therefore, a strong physical interaction between AQP4 and GLT1 is unlikely.

### GLT1 Expression Levels Are Not Altered in AQP4 Knockout Mice

**Figure 3.**
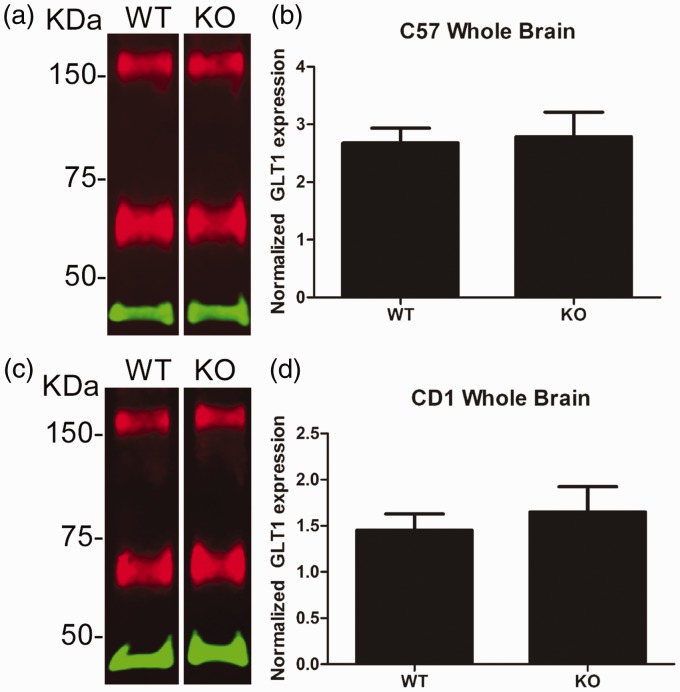
Glutamate transporter-1 (GLT1) protein expression in whole brain tissue from C57BL/6 and CD1 wild-type and aquaporin-4 (AQP4) knockout mice. (a) Representative Western blot of GLT1 (red) and β-actin (green) protein in whole brain tissue from C57BL/6 wild-type (WT) and AQP4 knockout (KO) mice. (b) Quantification of whole brain tissue from C57BL/6 WT (*n* = 6) and KO (*n* = 6) mice. (c) Representative Western blot of GLT1 (red) and β-actin (green) protein in whole brain tissue from CD1 wild-type (WT) and AQP4 knockout (KO) mice. (d) Quantification of whole brain tissue from CD1 WT (*n* = 6) and KO (*n* = 5) mice. KDa = kilodaltons.Data presented as mean ± *SEM*.

It is possible, however, for proteins to have a weak interaction that was not detected in our conditions. Therefore, we hypothesized that if GLT1 does interact with AQP4, then GLT1 levels may be slightly downregulated in AQP4^−/−^ mice. We measured GLT1 protein levels in wild-type and AQP4^−/−^ mice, but found no significant difference in whole brain tissue in either C57BL/6 or CD1 AQP4^−/−^ mice compared with their wild-type counterparts (Figure 3).

**Figure 4.**
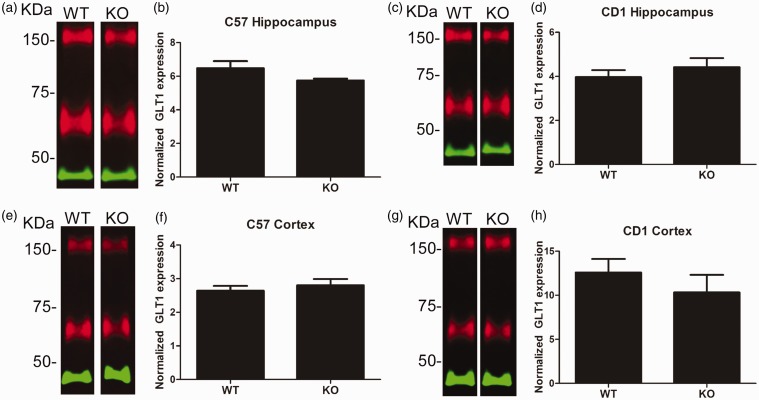
Glutamate transporter-1 (GLT1) protein expression in hippocampal and cortical tissue from wild-type and aquaporin-4 knockout (AQP4) knockout mice. (a) Representative Western blot of GLT1 (red) and β-actin (green) protein in hippocampal tissue from C57BL/6 wild-type (WT) and AQP4 knockout (KO) mice. (b) Quantification of hippocampal tissue from C57BL/6 WT (*n* = 7) and KO (*n* = 7) mice. (c) Representative Western blot of GLT1 (red) and β-actin (green) protein in hippocampal tissue from CD1 WT and AQP4 KO mice. (d) Quantification of hippocampal tissue from CD1 WT (*n* = 5) and KO (*n* = 5) mice. (e) Representative Western blot of GLT1 (red) and β-actin (green) protein in cortical tissue from C57BL/6WT and AQP4 KO mice. (f) Quantification of cortical tissue from C57BL/6 WT (*n* = 7) and KO (*n* = 7) mice. (g) Representative Western blot of GLT1 (red) and β-actin (green) protein in cortical tissue from CD1 WT and AQP4 KO mice. (h) Quantification of cortical tissue from CD1 WT (*n* = 5) and KO (*n* = 5) mice. KDa = kilodaltons. Data presented as mean ± *SEM*.

**Figure 5.**
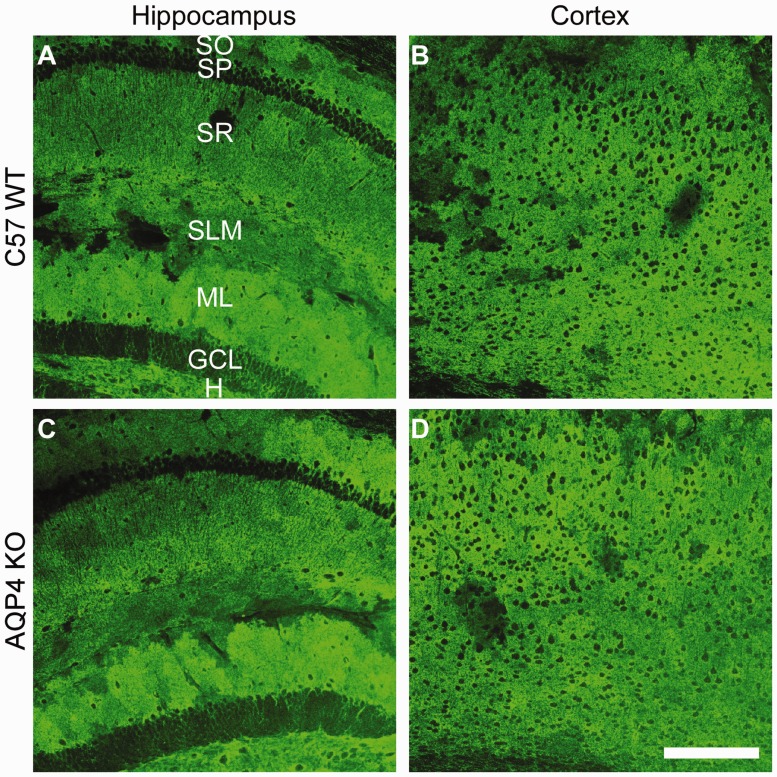
Glutamate transporter-1 (GLT1) immunoreactivity in C57BL/6 wild-type aquaporin-4 (AQP4) knockout mice. 20 × images of GLT1 immunoreactivity in C57BL/6 wild-type (a, b) and AQP4 knockout (c, d) mice. Representative images of the hippocampus (a, c) and cortex (b, d) are shown. Scale bar = 200 µm. For each group, two slices from each animal (*n* = 3) were used. SO = stratum oriens; SP = stratum pyramidale; SR = stratum radiatum; SLM = stratum lacunosum moleculare; ML = molecular layer; GCL = granule cell layer; H = hilus.

**Figure 6.**
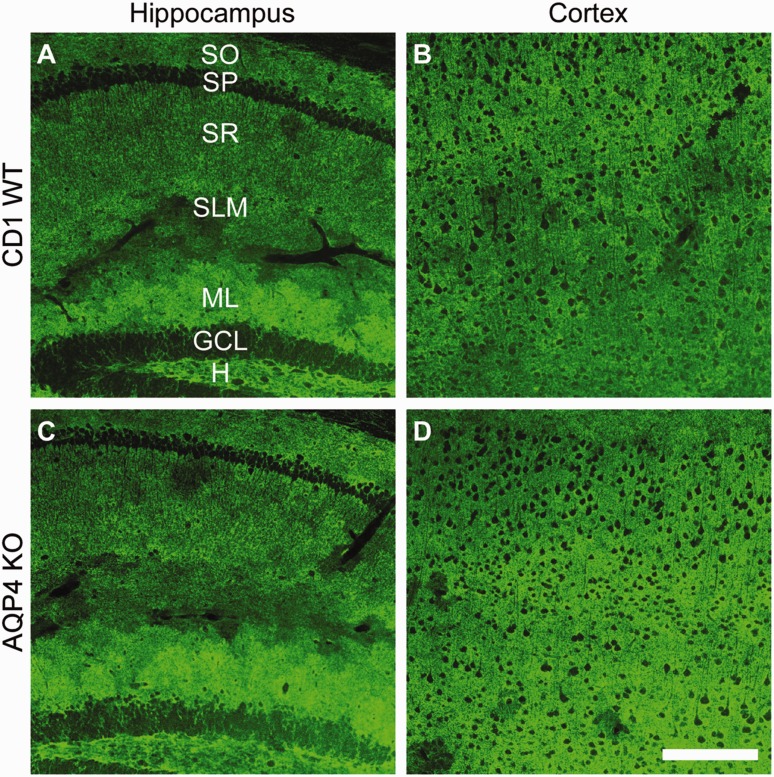
Glutamate transporter-1 (GLT1) immunoreactivity in CD1 wild-type and aquaporin-4 (AQP4) knockout mice. 20 × images of GLT1 immunoreactivity in CD1 wild-type (a, b) and AQP4 knockout (c, d) mice. Representative images of the hippocampus (a, c) and cortex (b, d) are shown. Scale bar = 200 µm. For each group, two slices from each animal (*n* = 3) were used. SO = stratum oriens; SP = stratum pyramidale; SR = stratum radiatum; SLM = stratum lacunosum moleculare; ML = molecular layer; GCL = granule cell layer; H = hilus.

We next decided to look for region-specific downregulation of GLT1. Since GLT1 is the predominant glutamate transporter in the forebrain, we microdissected out hippocampal and cortex tissue and analyzed it for GLT1 levels. We found no changes in hippocampal or cortex levels of GLT1 in either C57BL/6 or CD1 AQP4^−/−^ mice compared with C57BL/6 wild-type animals (Figure 4). This was confirmed with immunohistochemistry demonstrating no changes in GLT1 localization patterns in the hippocampus or cortex of C57BL/6 AQP4^−/−^ mice (Figure 5, Supplemental Figure 1). Similarly, no change in GLT1 immunoreactivity (Figure 6, Supplemental Figure 2) was observed in the hippocampus or cortex of CD1 AQP4^−/−^ mice compared with wild-type mice. In both C57BL/6 and CD1 mice, the laminar specificity and cellular localization of GLT1 appears unaltered throughout the hippocampus in the AQP4 knockout condition. GLT1 expression remains predominately absent in the neuronal layers (pyramidal and granule cell layers) but is highly abundant in other “astrocytic” layers, such as the molecular layer.

### AQP4 and GLT1 Colocalize in a Region-Specific Manner

**Figure 7.**
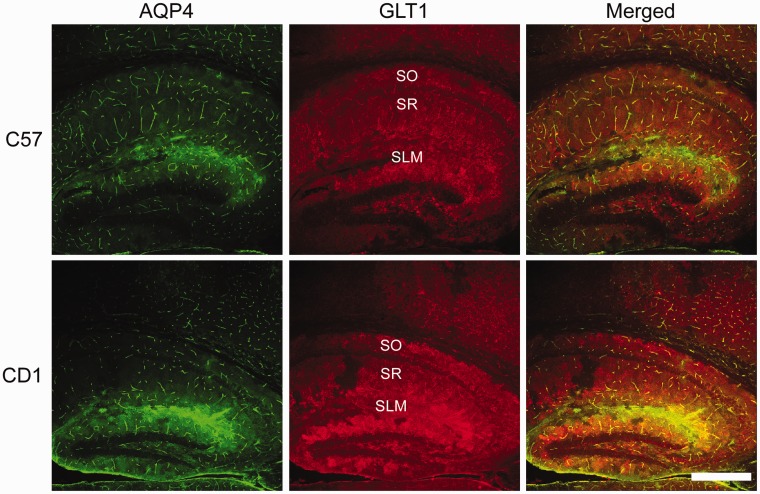
Aquaporin-4 (AQP4) and glutamate transporter-1 (GLT1) immunoreactivity in C57BL/6 and CD1 wild-type mice. 10 × images of AQP4 (green), GLT1 (red), and merged immunoreactivity of the hippocampus of C57BL/6 (top panels) and CD1 (bottom panels) wild-type mice. Scale bar = 200 µm. SLM = stratum lacunosmum moleculare; SO = stratum oriens; SR = stratum radiatum.

**Figure 8.**
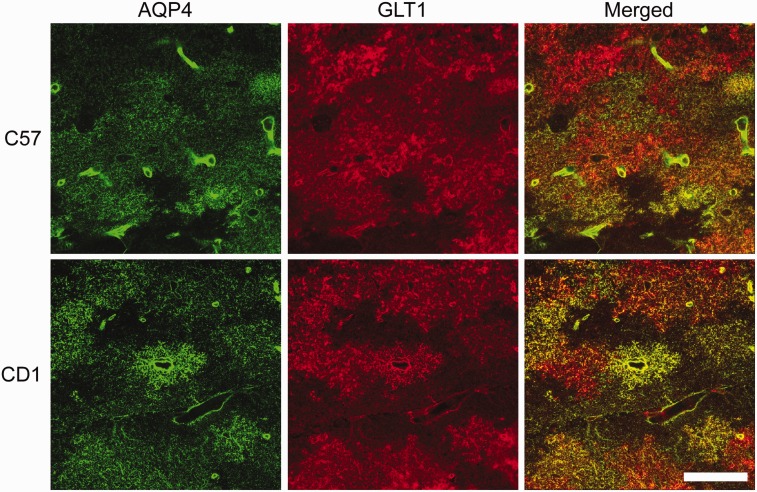
Aquaporin-4 (AQP4) and glutamate transporter-1 (GLT1) immunoreactivity in the stratum lacunosum moleculare of the hippocampus of C57BL/6 and CD1 wild-typemice. 63 × images of AQP4 (green), GLT1 (red), and merged immunoreactivity in the stratum lacunosum moleculare layer of the hippocampus from C57BL/6 (top panels) and CD1 (bottom panels) wild-type mice. Although AQP4 and GLT1 are highly colocalized (seen as yellow in the Merged images), there are patches of GLT1 immunoreactivity that lack AQP4 immunoreactivity. Scale bar = 50 µm.

**Figure 9.**
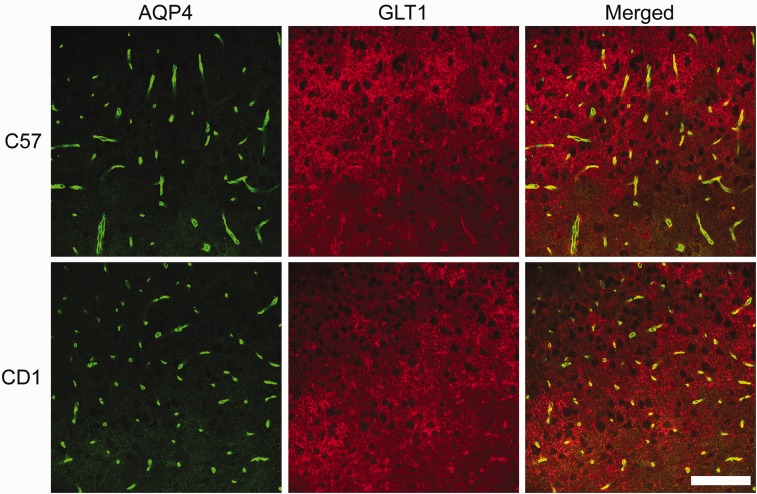
Aquaporin-4 (AQP4) and glutamate transporter-1 (GLT1) immunoreactivity in the cortex of C57BL/6 and CD1 wild-type mice. 40 × images of AQP4 (green), GLT1 (red), and merged immunoreactivity in the cortex of C57BL/6 (top panels) and CD1 (bottom panels) wild-type mice. AQP4 is highly expressed on astrocyte end-feet surrounding capillaries and GLT1 is abundantly found on cell membranes. Scale bar = 100 µm.

Finally, we examined both AQP4 and GLT1 immunoreactivity to determine whether any colocalization exists between these two proteins in the hippocampus of C57BL/6 and CD1 mice (Figure 7). GLT1 is highly expressed throughout the mouse hippocampus whereas AQP4 is largely found on astrocyte end-feet surrounding capillaries and in the stratum lacunosum moleculare (SLM) layer of the hippocampus. AQP4 and GLT1 are highly colocalized in the SLM of both C57BL/6 and CD1 mice but lack colocalization in other layers of the hippocampus such as such as the stratum oriens and stratum radiatum. A small portion of the cortex can also be seen, demonstrating AQP4 and GLT1 coexpression again on astrocyte end-feet surrounding capillaries but only GLT1 cellular membrane expression throughout the cortex. Higher magnification images confirmed abundant colocalization of AQP4 and GLT1 in the SLM layer of the hippocampus, although patches of GLT1-positive, AQP4-negative staining can be seen (Figure 8). In the cortex, AQP4 is almost exclusively found on astrocyte end-feet surrounding capillaries whereas GLT1 is expressed throughout (Figure 9). Therefore, AQP4 and GLT1 primarily lack colocalization in the cortex.

## Discussion

Here, we used coimmunoprecipitation, Western blotting, and immunohistochemistry to determine (a) whether interaction between GLT1 and AQP4 exists; and (b) whether GLT1 levels are altered in mice lacking AQP4. We found that in conditions of 1% NP-40 lysis buffer, GLT1 and AQP4 do not co-Ip in either CD1 or C57BL/6 whole brain lysates, suggesting the lack of a strong physical interaction between the two proteins. A weak interaction, however, is still possible and cannot be ruled out. We also found no alterations in GLT1 expression in the whole brain, hippocampus, or cortex of wild-type and AQP4^−/−^ mice. Furthermore, we observed region-specific colocalization of GLT1 and AQP4, rather than complete co-localization throughout the brain. Taken together, these data suggest the lack of any strong physical interaction between AQP4 and GLT1.

Several other studies have found co-localization between AQP4 and GLT1 in various regions of the brain and spinal cord (Hinson et al., 2008; Lee et al., 2013; Yang et al., 2013; Mogoanta et al., 2014), but only one study has conducted a co-IP between AQP4 and GLT1. Hinson et al. (2008) solubilized GFP-labeled AQP4-transfected cells and probed the lysates with an antibody to GLT1; AQP4 was found to co-Ip with GLT1 (Hinson et al., 2008). This is in direct contrast to our results demonstrating a complete lack of interaction between these two proteins. Our approaches, however, were very different. Although we both collected lysates in a 1% NP-40 lysis buffer, here, we used tissue from both CD1 and C57BL/6 adult mice whereas Hinson et al. (2008) used *in vitro* techniques, specifically looking at HEK-293 cells transfected with a GFP-AQP4 construct. It is possible, however, that a weak interaction between AQP4 and GLT1 exists that was not detectable in my co-IP. Arguing against this interpretation is (a) the robust ability to immunoprecipitate both GLT1 and AQP4 with the antibodies used, (b) the robust ability to detect supernatant GLT1 and AQP4 under the same conditions, and (c) the predominate lack of co-localization examined throughout the mouse brain.

A strong physical interaction between AQP4 and GLT1 is unlikely even in the diseased brain. In the intrahippocampal kainic acid (IHKA) model of epilepsy, AQP4 and GLT1 exhibit different regulation patterns (Hubbard et al., 2016). Within 1 day of IHKA injections, dorsal hippocampal GLT1 expression is upregulated whereas AQP4 is downregulated. By seven days post IHKA injections, GLT1 is drastically downregulated. At this same time point, AQP4 dorsal protein expression is near control levels. Furthermore, AQP4 mRNA is upregulated after IHKA injections whereas GLT1 mRNA is largely unaffected (Hubbard et al., 2016). Therefore, it is unlikely that the diseased state induces an association between AQP4 and GLT1.

Although no other studies have reported on the physical interaction between AQP4 and GLT1, several other studies have examined GLT1 expression levels in AQP4^−/−^ mice. Specifically, a reduction in GLT1 protein levels was reported in primary astrocyte cell cultures from cerebral cortices of wild-type and AQP4^−/−^ mice (Zeng et al., 2007). GLT1 protein expression was reduced by less than 20% in spinal cord tissue (Chen et al., 2010) and by nearly 30% in the cerebellum (Yan et al., 2013) of AQP4^−/−^ mice. A drastic reduction of GLT1 protein levels (∼50%) was reported in the amygdala of AQP4^−/−^ mice (Li et al., 2012). AQP4^−/−^ mice clearly exhibited a region-specific reduction of GLT1 expression. In direct contrast to our findings, a 20% to 35% reduction in GLT1 hippocampal levels in AQP4^−/−^ mice has been reported (Yan et al., 2013; Yang et al., 2013; Kong et al., 2014). Similarly, cortical regions of AQP4^−/−^ mice exhibited a 14% to 26% reduction of GLT1 expression (Wu et al., 2008; Yan et al., 2013). Further studies will be needed to clarify these discrepancies.

It has been hypothesized that a downregulation of GLT1 may be partially responsible for the impaired synaptic plasticity observed in AQP4^−/−^ mice (Skucas et al., 2011; Li et al., 2012; Szu and Binder, 2016). Our findings, however, suggest that GLT1 levels are fully intact in AQP4^−/−^ mice. Therefore, impairments such as difficulties in learning and memory formation in AQP4^−/−^ mice cannot be accounted for by reduced GLT1-dependent glutamate clearance. As suggested by Skucas et al. (2011), synaptic plasticity deficits in AQP4^−/−^ mice may be instead due to neurotrophin dysregulation (Skucas et al., 2011). Specifically, AQP4 may be important to the regulation of the low-affinity neurotrophin receptor p75^NTR^, which was downregulated in AQP4^−/−^ mice (Skucas et al., 2011).

Aquaporin-4 plays a role in regulating extracellular space (ECS) volume (Binder et al., 2004; Yao et al., 2008). Specifically, AQP4^−/−^ mice have an increase in ECS volume with no difference in tortuosity (Yao et al., 2008). AQP4^−/−^ mice also have delayed clearance of extracellular K^+^ (Amiry-Moghaddam et al., 2003; Binder et al., 2006; Haj-Yasein et al., 2015). The uptake of K^+^ into astrocytes after neuronal activity produces a change in the osmotic driving force in favor of water uptake into astrocytes (Jin et al., 2013). This uptake causes astrocytes to swell, thus reducing the ECS volume. With an increased ECS volume, such as in AQP4^−/−^ mice, a reduced accumulation of K^+^ may be observed after a mild stimuli and may not alter astrocyte swelling (Jin et al., 2013; Anderova et al., 2014). After neuroexcitation or more severe stimuli, such as hypoosmotic stress, oxygen glucose deprivation, or high K^+^, reduced water permeability causes slowed ECS contraction (Haj-Yasein et al., 2012; Jin, Zhang, Binder, and Verkman, 2013; Anderova et al., 2014) and consequently may alter the electrochemical driving force for K^+^ reuptake by astrocytes in AQP4^−/−^ mice (Jin et al., 2013).

Altered ECS dynamics may explain the different disease courses seen in AQP4^−/−^ mice, such as in models of edema and epilepsy. AQP4 deficiency can be neuroprotective in cytotoxic edema but leads to a worsened prognosis (impaired water removal through glia limitans) in vasogenic edema (Papadopoulos et al., 2004). Similarly, AQP4^−/−^ mice had a higher seizure threshold but prolonged seizure duration (Binder et al., 2006) and increased number of seizures (Lee et al., 2012) in models of epilepsy. While the increased ECS volume may increase the threshold to seizure generation (Binder et al., 2004), slowed deswelling of astrocytes could lead to prolonged and more severe seizures (Binder et al., 2006). Therefore, perturbed extracellular space volume dynamics and consequently altered ion and neurotransmitter clearance, not reduced GLT1 expression, may offer an alternative explanation to differences in disease progression seen in AQP4^−/−^ mice.

## Author Contributions

J. A. H. prepared the manuscript and performed all experiments. D. K. B. contributed oversight to the experimental design and assisted in preparation of the manuscript.

## Supplementary Material

Supplementary material
