# Supplementary material for: Unaltered Glutamate Transporter-1 Protein Levels in Aquaporin-4 Knockout Mice
Source: ASN Neuro. 2017 Jan 1;9(1):1759091416687846. doi: 10.1177/1759091416687846 (PMC5315234; doi:10.1177/1759091416687846)
Supplement: Supplementary material [file ASN687846_supplementary_figures.pdf]

Supplemental Figures

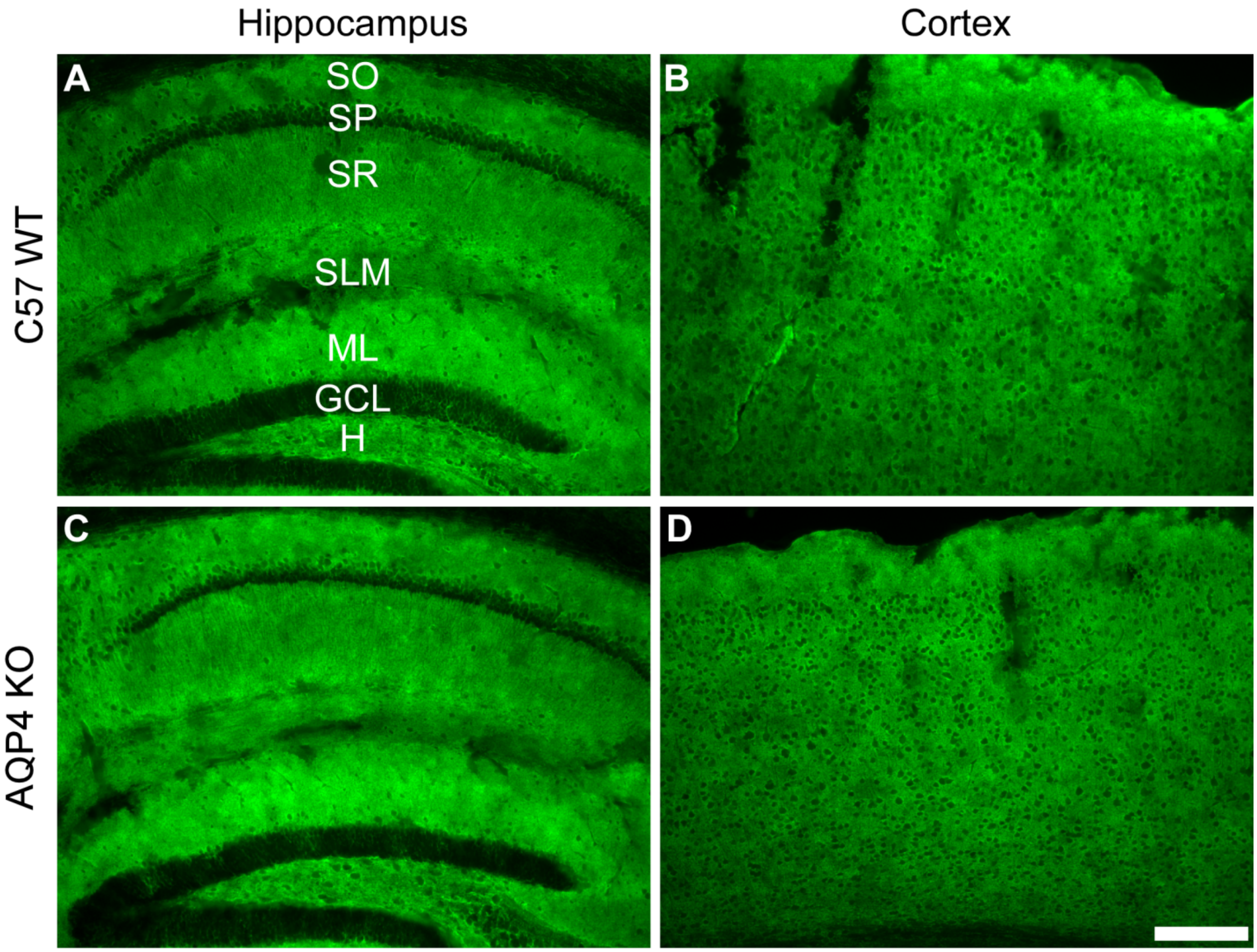

Supplemental Figure 1. Glutamate transporter-1 (GLT1) immunoreactivity in C57BL/6 wild-type aquaporin-4 (AQP4) knockout mice. 10x images of GLT1 immunoreactivity in C57BL/6 wild-type **(A, B)** and AQP4 knockout **(C, D)** mice. Representative images of the hippocampus **(A, C)** and cortex **(B, D)** are shown. Scale bar = 200 μm. For each group, 2 slices from each animal (n = 3) were used. SO = stratum oriens; SP = stratum pyramidale; SR = stratum radiatum; SLM = stratum lacunosum moleculare; ML = molecular layer; GCL = granule cell layer; H = hilus.

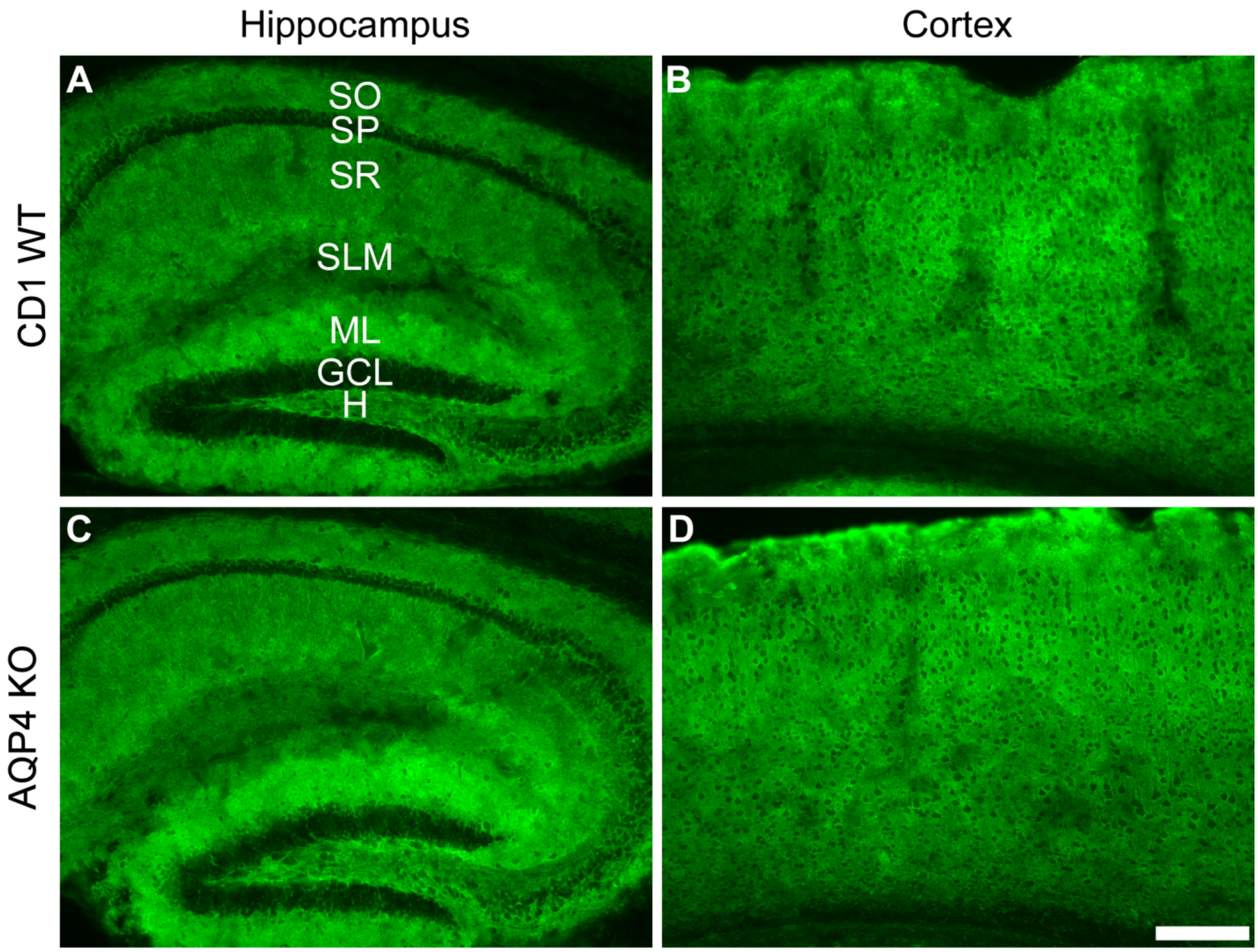

Supplemental Figure 2. Glutamate transporter-1 (GLT1) immunoreactivity in CD1 wild-type and aquaporin-4 (AQP4) knockout mice. 10x images of GLT1 immunoreactivity in CD1 wild-type **(A, B)** and AQP4 knockout **(C, D)** mice. Representative images of the hippocampus **(A, C)** and cortex **(B, D)** are shown. Scale bar = 200 µm. For each group, 2 slices from each animal (n = 3) were used. SO = stratum oriens; SP = stratum pyramidale; SR = stratum radiatum; SLM = stratum lacunosum moleculare; ML = molecular layer; GCL = granule cell layer; H = hilus.
